# GDF11 and aging biology - controversies resolved and pending

**DOI:** 10.20517/jca.2023.23

**Published:** 2023-10-20

**Authors:** Laura Ben Driss, John Lian, Ryan G. Walker, James A. Howard, Thomas B. Thompson, Lee L. Rubin, Amy J. Wagers, Richard T. Lee

**Affiliations:** 1 Department of Stem Cell and Regenerative Biology and the Harvard Stem Cell Institute, Harvard University, Cambridge, MA 02138, USA.; 2 Department of Molecular and Cellular Biosciences, University of Cincinnati, Cincinnati, OH 45267, USA.; 3 Department of Pharmacology and Systems Physiology, University of Cincinnati, Cincinnati, OH 45267, USA.; 4 Stanley Center for Psychiatric Research, Broad Institute of MIT and Harvard, Cambridge, MA 02142, USA.; 5 Paul F. Glenn Center for the Biology of Aging, Harvard Medical School, Joslin Diabetes Center, Boston, MA 02115, USA.; 6 Cardiovascular Division, Department of Medicine, Brigham and Women’s Hospital, Boston, MA 02115, USA.

**Keywords:** GDF11, aging, systemic factor, heart, skeletal muscle, brain

## Abstract

Since the exogenous administration of GDF11, a TGF-ß superfamily member, was reported to have beneficial effects in some models of human disease, there have been many research studies in GDF11 biology. However, many studies have now confirmed that exogenous administration of GDF11 can improve physiology in disease models, including cardiac fibrosis, experimental stroke, and disordered metabolism. GDF11 is similar to GDF8 (also called Myostatin), differing only by 11 amino acids in their mature signaling domains. These two proteins are now known to be biochemically different both *in vitro* and *in vivo*. GDF11 is much more potent than GDF8 and induces more strongly SMAD2 phosphorylation in the myocardium compared to GDF8. GDF8 and GDF11 prodomain are only 52% identical and are cleaved by different Tolloid proteases to liberate the mature signaling domain from inhibition of the prodomain. Here, we review the state of GDF11 biology, highlighting both resolved and remaining controversies.

## Graphical Abstract

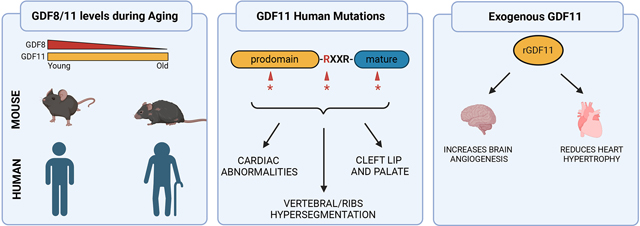

## INTRODUCTION

Aging plays an important role in almost all the most prevalent diseases afflicting modern humans. While there are “hallmark” mechanisms in the aging of many organisms^[1]^, the molecular intersections of aging biology and human disease remain mysterious. As an example, one area of intersection is fibrosis and inflammation, both of which are associated with aging. In all tissues, an inflammatory response to damaged tissues provides key molecular signals for the activation of reparative cells^[2]^. Activation of this post-injury inflammatory response is necessary to promote adhesive interactions between leukocytes and endothelial cells and to drive the infiltration of these leukocytes to clear dead cells and initiate repair. To escape potential deleterious damage due to excessive inflammation, activation of an anti-inflammatory response is also essential. In parallel, tissue-resident fibroblasts proliferate and transdifferentiate into myofibroblasts and deposit extracellular matrix to restore and protect the structural integrity of the tissue. Among the many molecular pathways implicated in fibrosis and repair, members of the TGF-ß superfamily appear central. For example, TGF-ß family ligands participate in cardiac remodeling after infarction via regulation of inflammation and repair^[3]^ and excessive TGF-ß signaling can also lead to cardiac fibrosis^[4]^. In many tissues, blockade of TGF-ß signaling is sufficient to blunt fibrotic responses, but genetic abolition of this signaling system, achieved by targeted disruption of its receptor or essential signal transducing proteins, has significant pathological consequences, including possible induction of systemic inflammation and a heightened risk of cancer^[5]^.

**Figure 1.**
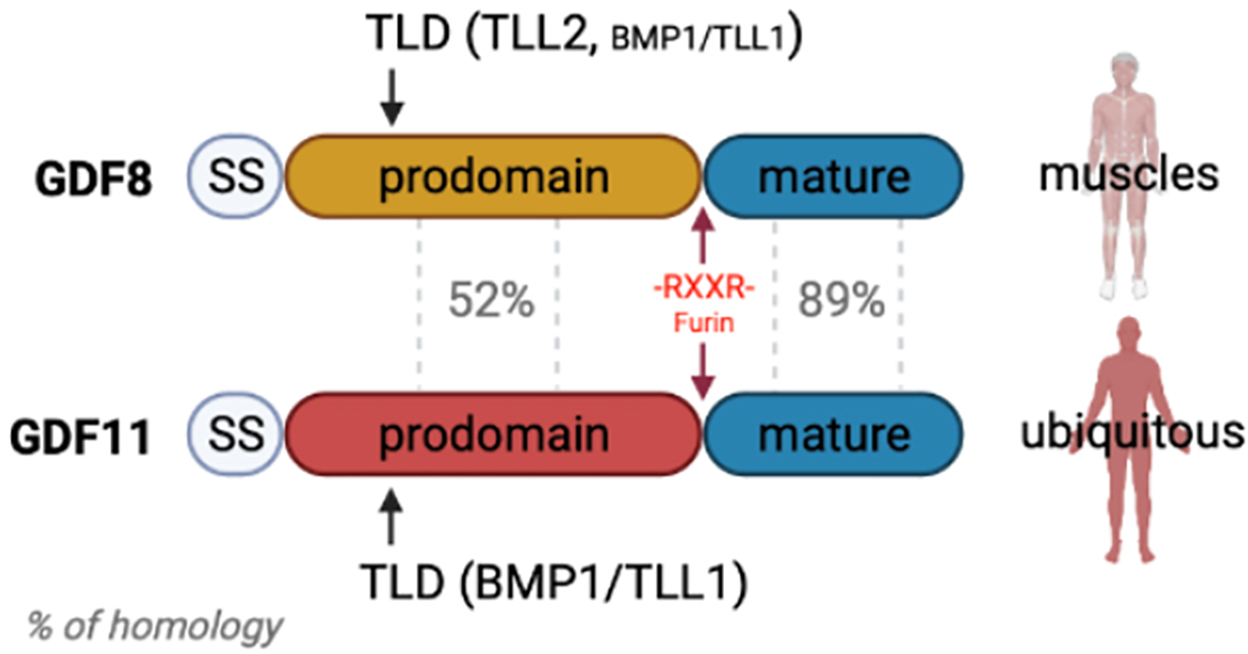
Distinct structure and expression patterns of GDF8 and GDF11. GDF8 and GDF11 share 89% amino acid identity in their mature domain, but only 52% in their prodomain. During processing, the signal sequence (SS) is removed, and the pre-pro-ligands are cleaved by Furin and Tolloid proteases (TLDs) to prepare the mature ligand for future signaling. GDF8, expressed predominantly in skeletal muscle, is cleaved by all TLDs and preferentially by TLL2 due to the availability of this TLD in the muscle, while the ubiquitously expressed GDF11 is cleaved preferentially by BMP1 and TLL1^[6]^. Figure made by @Biorender.

In this review, we focus on GDF11, a member of the TFG-ß superfamily known to be required for the development of many organs but also suggested to have additional adult mammalian roles in the cardiovascular, musculoskeletal, and neurovascular systems. We discuss the molecular properties of GDF11 as well as its closely related ligand GDF8, also called Myostatin, and their reported effects and functions in regenerative tissues and aging diseases [Figure 1]. Over the past decade, several controversies have arisen around GDF11, but many of the major controversies have been systematically addressed. In addition, the biology of GDF11 is clearly relevant to humans given new reports of GDF11 loss-of-function genetic diseases that can affect the cardiovascular system, musculoskeletal, and nervous systems^[6]^.

## GDF11: BASIC BIOLOGY, STRUCTURE AND FUNCTIONS

### Systemic factors during aging

Aging is associated with a decline of tissue and organ functions due to diverse biological processes including oxidative damage, mitochondrial dysfunction, and genome instability^[7]^. The regenerative capacity of some tissues, such as the brain or the skeletal muscles, may decline with aging in part due to a diminution of stem/progenitor cell numbers or dysregulation of stem cell responses. In adult mammals, new cardiomyocytes are formed from pre-existing cardiomyocytes rather than differentiating from a stem cell pool, but this capacity also declines with age in mammals including humans, and most adult cardiomyocytes appear to have limited ability to proliferate after mid-life^[8]^. The molecular mechanisms underlying the decline in cardiogenesis with aging in mammals are unclear, but, interestingly, voluntary exercise can restore youthful rates of new cardiomyocyte formation in old mice^[9]^. This shows that the age-dependent loss of new cardiomyocyte formation is reversible. The molecular mechanisms of cardiomyocyte aging remain mysterious, but as suggested by these exercise data, it is possible that systemic effects could be important.

Systemic mechanisms for organ aging have revealed multiple pathways, but unsurprisingly, no single mechanism. For example, declining skeletal muscle regenerative capacity with age is in part due to a defect of NOTCH pathway activation^[10]^. This age-related repair defect could be rescued after an injury in old mice by injection of a NOTCH activator directly in the muscle, to force NOTCH pathway activation. Moreover, heterochronic transplantation experiments in which old muscles were grafted into young hosts, and vice versa, showed that old muscles in a young environment can regenerate after an injury while young muscles in an old environment regenerate no better than old muscles in an old host. These results indicated that the poor muscle regenerative capacity of old mice is due to the muscle environment^[11]^. One possible explanation for this observation, which has been under intense investigation for the past two decades, is that blood-borne circulating factors play a key role in regulating tissue regenerative responses and that variations in the level and/or activity of these factors with age are a key determinant of the observed differences in repair activity.

The aging process induces different structural and functional changes in the body suggesting changes between young and old subjects. To understand the possible influence of systemic factors in aging phenotypes, many studies have used parabiosis experiments. This technique consists of joining two mice surgically to share a common circulation. When an old mouse and a young mouse are joined in this manner, it is called heterochronic parabiosis; the old mice are exposed to factors present in young blood, and the young mice to factors present in old blood. Soluble factors and cells cross-circulate in heterochronic parabiotic mice, and many studies^[12–18]^, demonstrated that this intervention can restore more youthful functions in the heart, skeletal muscle, bone, endocrine, and central nervous systems of old partners^[12–23]^. Conversely, heterochronic circulation has been shown to suppress healthy function in young animals in a subset of these systems^[19–21,24]^. Rando’s lab showed that heterochronic parabiosis restores the activation of the NOTCH signaling pathway and improves skeletal muscle regeneration and stem cell activation in the old mice^[17]^ but also induces heightened WNT signaling and fibrosis that suppresses stem cell function and regenerative myogenesis in the young mice^[25]^. Thus, in skeletal muscle, heterochronic parabiosis exerts clear bi-directional effects, remodeling muscle ultrastructure and enhancing repair in aged partners^[12,13,17]^, and suppressing regenerative function and exacerbating fibrosis in young partners^[19]^. These results highlight the importance of blood-circulating factors during aging; vascular-active circulating factors in the context of aging and the brain have been recently reviewed by Bieri *et al.*^[26]^.

### GDF11 is a circulating factor that reduces cardiac hypertrophy in aged mice

Over the past 10 years, many studies, have sought to identify candidate molecular geronic (aging-related) factors, with studies implicating various hormones, cytokines, growth factors, and immune regulatory proteins^[26–29]^. Heterochronic parabiosis was shown to reduce cardiac hypertrophy in old mice without a concomitant increase in heart size in young mice^[15]^. In that study, the effect of heterochronic parabiosis on the old heart was also apparent in the size of the cardiomyocytes themselves. An additional experiment was performed as a control, in which the mice were surgically connected but did not share a common cross-circulation. In these experiments, the reduction in size of the old heart was not observed in the absence of a shared circulation suggesting that young blood contains factors that regulate cardiac size in old mice. Taking a proteomics approach, 13 possible candidates were identified that significantly varied between young and old mice, but the study focused on GDF11 as it is a TGF-ß family member that is closely related-protein GDF8 was already known to regulate hypertrophy in the heart. Then a later study demonstrated that delivery of recombinant GDF11 (rGDF11) to old mice led to a decreased heart size^[15]^. An important point is that these experiments used an aptamer and monoclonal antibody against GDF11 that we believed at the time to be specific for GDF11, but both were later shown to cross-react with GDF8^[30]^. Thus, our experiments erroneously concluded that GDF11 levels decline in mouse blood with age, when in fact, our reagents were measuring both GDF11 and GDF8. Given the substantially greater abundance of GDF8 in circulation (50–100 times that of GDF11), it is not surprising that when reagents and assays capable of discriminating GDF11 from GDF8 eventually became available, they showed that GDF8 (not GDF11) was the age-dependent ligand^[31]^ (and Kato *et al.*, under review). Nonetheless, the effects of exogenous GDF11 supplementation do appear clear and age-dependent.

An initial report showed that administration of exogenous GDF11 at 0.1 mg/kg to aged mice reduced cardiac hypertrophy, like the heterochronic parabiosis experiments^[15]^. But then, another lab performed a study in 2015 that did not reproduce this finding at 0.1 mg/kg with well-characterized recombinant GDF11^[32]^. During this time, quality control studies were performed and found that commercial preparations of GDF11 varied in protein quantity, and this was likely to be important when using GDF11 as an *in vivo* agent. This information was communicated to the companies that manufactured GDF11 and publicly stated that dose and protein quality may affect results. The Houser lab then showed, in 2016, with careful dose-response experiments that exogenous administration of GDF11 significantly reduced TAC-induced cardiac hypertrophy and improved cardiac function in a dose-dependent fashion^[33]^. However, mice receiving high doses of GDF11 (5 mg/kg) developed cachexia and premature death, showing that excessively high doses of GDF11 can cause deleterious effects at high doses, as others have confirmed^[30,34,35]^.

### Basic biology of GDF8 and GDF11

GDF11, also called Bone Morphogenetic Protein 11 (BMP11), and its closely related protein GDF8, also known as Myostatin, are members of the TGFβ superfamily and share 89% of identity. GDF8 is only expressed in skeletal muscle and plays an evolutionarily conserved role in postnatal skeletal muscle growth, limiting both the number and size of individual muscle fibers^[36]^. Deletion of the Gdf8 gene or inhibition of GDF8 protein leads to muscle hypertrophy in many mammals and fish^[37–39]^. GDF11, in contrast, is ubiquitously expressed and plays different roles during mammalian development, regulating anterior/posterior patterning, and different tissue formations such as kidneys, endocrine pancreas, spleen stomach, and olfactory neurogenesis^[40–45]^. Due to the perinatal lethality of Gdf11-knockout mice^[40,41]^, which exhibit homeotic skeletal transformations, cleft palate, and renal agenesis, the functions of GDF11 in postnatal tissues are less explored.

GDF8 and GDF11 are produced as unprocessed pre-pro complex proteins, and different cleavages are required to separate the mature signaling domain from the tight binding of the inhibitory prodomain. The critical cleavage of the prodomain is made by the Tolloid proteases (TLDs), which are zinc-dependent metalloproteinases that include 4 members: bone morphogenetic protein 1 (BMP1), mammalian tolloid (mTLD), tolloid-like 1 (TLL1) and TLL2. TLD substrates are wide-ranging and are essential for tissue patterning and extracellular matrix assembly. GDF8 is cleaved by the four members of the TLD family, preferentially by TLL2, whereas GDF11 is cleaved by BMP1 and TLL1. *In vitro* experiments showed that a TLD cleavage-resistant mutation in the prodomain prevents ligand activation. *In vivo*, administration of a mutant GDF8 prodomain that is resistant to TLD cleavage increased muscle mass as GDF8 inhibitors do, but wild-type GDF8 prodomain does not inhibit in this manner. Thus, TLD cleavage of the prodomain is essential for ligand activation.

The mature domains of GDF8 and GDF11 are disulfide-linked homodimers with a propeller-like shape. This arrangement creates symmetrical concave and convex surfaces which are used for receptor binding. To signal, ligands assemble a combination of two Type II and two Type I Ser/Thr kinase receptors that have extracellular ligand binding domains. This complex allows the Type II receptor to phosphorylate the Type I receptor and initiates the downstream SMAD signaling cascade. While there are over 30 TGFβ family ligands, only 5 Type II receptors and 7 Type I receptors are available for signaling. GDF11 and GDF8 are members of the Activin subclass which signals through ALK4, ALK5, and ALK7 members of the Type I receptors. Signaling is differentiated at the receptor level where different combinations of Type I and Type II receptors can elicit different downstream responses.

### Exogenous GDF11 improves brain vasculature

Aging is a well-known risk factor for many neurological disorders, including vascular dementia. With increasing age, there is a general loss in vascular density and blood flow, but also in the quality of the remaining vasculature^[46–48]^. There are also age-related changes in the blood-brain barrier, with a loss of endothelial junctional barrier proteins^[49]^, a reduction in specific transport from blood to brain, a reduced transport of material from the brain to blood, and an increase in non-specific blood-brain transport^[50]^. With these changes, increased brain inflammation, a hallmark of aging, affects vascular integrity, probably leading to the increased entry of immune cells into the brain^[51]^. Thus, aging encompasses a constellation of vascular changes, many of which are also observed in diseases of the central nervous system. Numerous proteomics-type studies of young and old serum have been performed, identifying multiple blood-borne factors that influence brain function negatively or positively^[14–17,20,52,53]^. The circulating factors identified thus far derive from a variety of peripheral tissues and are secreted into the blood to signal at a distance, in an endocrine fashion.

The beneficial effects of heterochronic parabiosis were described in the brains of old animals^[14,16,17,20,52,53]^. Young blood can stimulate an increase in neurogenesis in both the subventricular zone and the hippocampus^[14,16,20,24,52,53]^, associated with a greater vascular density in those regions. Accompanying these neural stem/progenitor and neuronal effects, the exposure to young blood led also to greater vascular density, not only in the neurogenic niches but throughout most of the brain. Brain endothelial cells appear to be particularly sensitive to heterochronic parabiosis, which may be expected as some circulating factors may not pass through the blood-brain barrier efficiently in the absence of injury^[14,54]^. Systemic administration of rGDF11 summarizes many positive effects of heterochronic parabiosis, including vascular remodeling of aged blood vessels and increased numbers of neural stem cells. rGDF11 also induces the proliferation of brain capillary endothelial cells and activates the SMAD2/3 pathway in these cells *in vitro*^[55]^. Although rGDF11 is unable to cross the blood-brain barrier, it likely binds to its receptors on endothelial cells, stimulating them to secrete pro-neurogenic factors^[56–58]^.

GDF11 is expressed in the developing mouse hippocampus, where it acts as an inhibitor of neurogenesis^[44,45,59]^. Lowering the expression of GDF11 in the adult hippocampus, using a Cre-inducible Gdf11 deletion mouse, increases the number of neural stem/progenitor cells^[60]^ confirming that endogenous GDF11 acts locally in the brain and acts as a negative regulator of neurogenesis even in adult mice. These results reveal that GDF11 can have different, and even opposite effects, when it is acting locally *vs*. hormonally, by targeting receptors on different cell types.

Potential therapeutic applications have arisen from studies of the effects of exogenous rGDF11 on the brain. An injection of rGDF11 twice per day, in Alzheimer’s disease model mice with APP/PSEN mutations led to improvement of the cognitive performance of these mice associated with reduced inflammation, increased number of brain endothelial cells, and increased blood flow^[61]^. Other studies have found that infusion of rGDF11 after brain ischemia/reperfusion reduced mortality, improved behavior, reduced gliosis and inflammation, increased staining for myelin basic protein, and increased angiogenesis^[62,63]^.

### The mature domains of GDF11 and GDF8 are similar but not equivalent

Because GDF8 and GDF11 mature domains differ only by 11 amino acids, it has long been assumed that the GDF11 and GDF8 ligands would signal similarly. Their activities are similar when studied with *in vitro* assays^[64]^ and in addition to their strong homology, these proteins are also inhibited by similar secreted proteins and bind to the same receptors^[64]^. Together, these data led to the previously popular concept that GDF8 and GDF11 were functionally interchangeable, with *in vivo* differences apparent due solely to variation in tissue-specific expression.

While it was shown that GDF8 and GDF11 signal through similar receptors, a direct rigorous comparison of the ligands had not been performed until we published a study demonstrating that GDF8 and GDF11 have significant differences in their signaling properties in multiple cell lines, showing that GDF11 is much more potent than GDF8. We also demonstrated that administration of GDF11 more potently induces SMAD2 phosphorylation in the myocardium compared to GDF8. A comparison of the GDF8 and GDF11 crystal structures revealed key structural differences between the two ligands and provided a potential basis as to why GDF11 is a more potent ligand than GDF8. To conclude, structural and biochemical experiments showed that GDF11 and GDF8 are not functionally equivalent, perhaps most importantly when ligand concentrations are low, as *in vivo*.

However, these studies did not address whether endogenous GDF8 and GDF11 are functionally equivalent *in vivo*. To assess this question, an *in vivo* study used different models in which the mature domains of GDF11 and GDF8 are genetically modified. They replace the entire mature domain of GDF8 with the mature domain of GDF11 (GDF8^GDF11MD^) to investigate the interchangeability of the two ligand signaling domains. In this model, the entire Gdf11 mature domain replaces the Gdf8 mature domain in the Gdf8 locus, yielding Gdf8^Gdf11MD^ mice entirely lacking GDF8 but with levels of GDF11 that are 25- to 50-fold higher than normal^[65]^. That showed that young Gdf8^Gdf11MD^ mice exhibit modest reductions in muscle mass in some but not all muscles, with no apparent impact on total body weight, muscle regenerative potential, bone development, cardiac size, and function, or survival^[65]^. A similar version of this genetic modification was reported also by another lab^[66]^, and both papers reported that chronically high levels of circulating GDF11 were surprisingly well tolerated in mice. In the same first study, two other mouse models were studied in which two specific amino acids of GDF11 were swapped with the corresponding GDF8 amino acids. That substitution of just two amino acids from GDF8 into GDF11 diminished GDF11 ligand potency and changed axial skeleton development. These substitutions resulted in a consistent phenotype with Gdf11-deficient mice, with axial skeletal defects without apparent perturbation of skeletal/cardiac muscle development or homeostasis. These combined experiments, uncover some distinctive features between the GDF11 and GDF8 mature domains *in vivo*, suggesting that the endogenous mature ligands are functionally different. Taken together, these findings provide direct evidence that structural and biochemical differences in the ligand mature signaling domains contribute significantly to their roles in mammalian development.

### Evidence for a “Triggered” prodomain-ligand state

**Figure 2.**
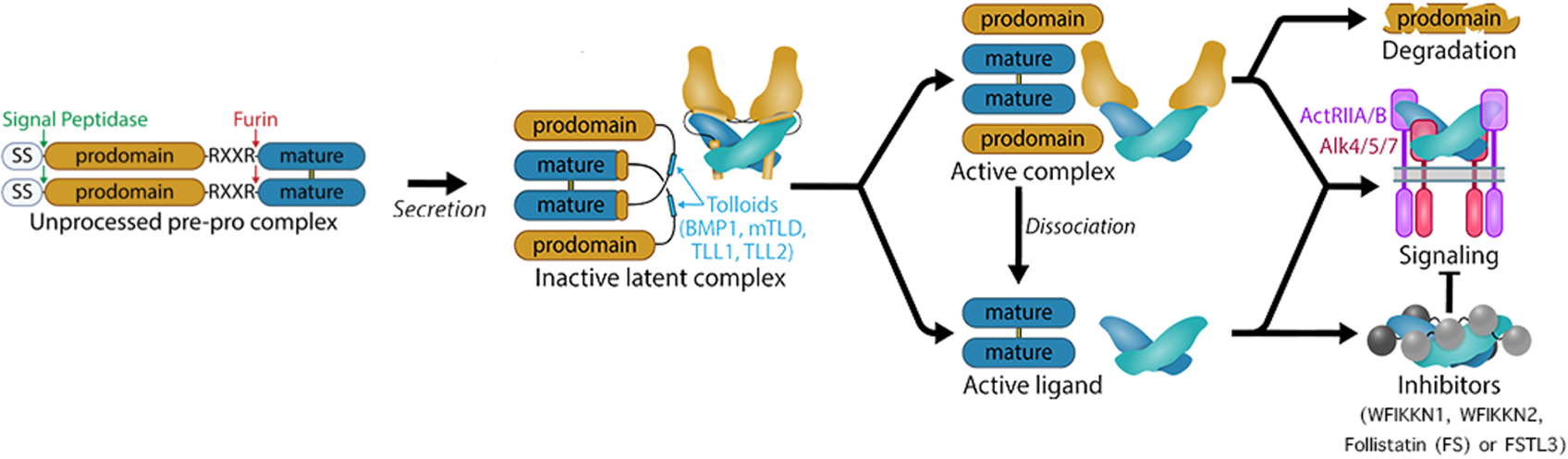
Proteolytic processing of GDF8 and GDF11 prodomains is a critical regulatory step. Proteolytic processing is necessary to pass from an unprocessed pre-pro complex protein to an active ligand able to signal. After a signal peptidase, the Furin protein recognizes and cleaves a specific motif -RXXR- between the prodomain and the mature domain. The inactive latent complex is then cleaved by the Tolloids family of protease to separate the prodomain from the active ligand. After cleavage, the prodomain is readily displaced when the mature ligand binds to the type II receptor and is likely degraded. Upon binding the type II receptor, a type I receptor is recruited and phosphorylated to activate downstream signaling pathways. The mature domain can also interact with inhibitors such as WFIKKN1, WFIKKN2, Follistatin (FS), or FSTL3.

After translation, both GDF8 and GDF11 are trapped in a non-signaling, latent complex by the N-terminal prodomain (termed the pro-complex) composed of a prodomain and a mature signaling domain. Activation of the pro-complex requires a second cleavage by TLD proteases at a highly specific location in the prodomain. The two pieces of the prodomain bind much weaker, allowing the mature ligand to be liberated and bind its receptors to signal. Thus, the synthesis of active GDF8 and GDF11 requires multiple steps. Step 1 - Two chains assemble and are connected in the mature region through a disulfide bond. Step 2 - The prodomain is cleaved from the mature domain via the protease Furin in the -RXXR- motif. Step 3 - The signal sequence is cleaved upon secretion of the pro-complex in the extracellular matrix. Step 4 - A TLD protease cleaves the prodomain, weakening its interaction with the mature ligand. Step 5 - The mature domain binds the cognate receptors and activates SMAD molecules. In addition to TLD, an activated state of the pro-complex can occur, where the prodomain is still attached to the ligand, but the ligand can signal without the addition of TLD [Figure 2]. This activated state is about 50% less active than the mature ligand alone suggesting that GDF8 and GDF11 exist in multiple states, ranging from the apo ligand which has the most activity to the latent pro-complex which has little to no activity. Because mass spectrometry measures total protein but not different molecular states, the different states in human blood are currently incompletely defined.

**Figure 3.**
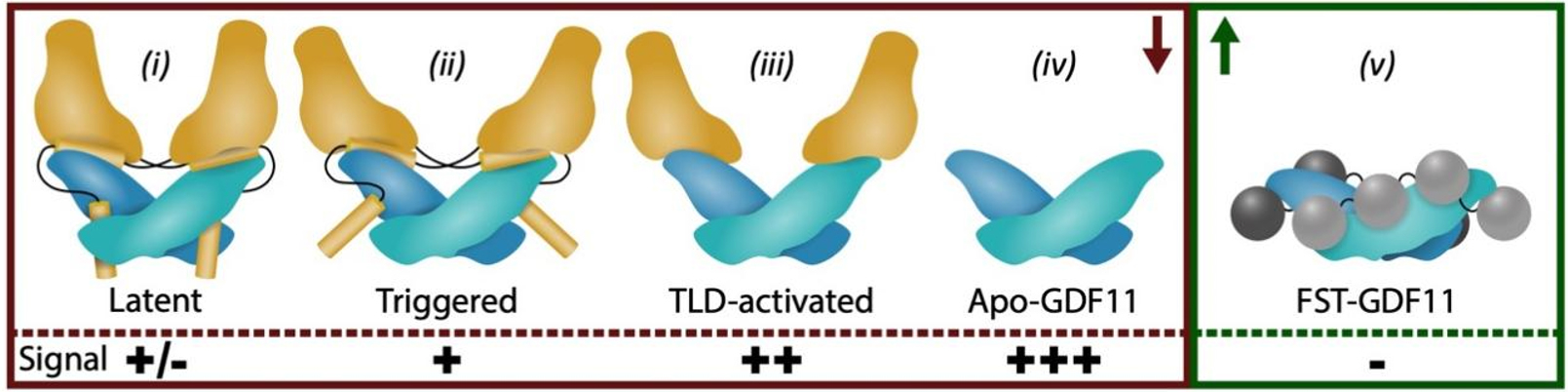
Different potential states of GDF11. (i) Latent, with green and purple dimer and brown prodomains; (ii) Triggered as realized through acid activation; (iii) Tolloid processed; (iv) free ligand; and (v) antagonist bound with red and pink antagonists. + denotes active signaling states. Arrows indicate the possibility that some specific forms may change with age.

While latent GDF11 and GDF8 are known to be activated by the Tolloid proteases [Figure 2], which cleave the prodomains, activation can also be achieved under acid conditions in what is referred to as “acid-activation”^[67]^. This process was originally thought to dissociate the prodomain from GDF8; however, purification of the acid-activated form revealed that the prodomain remained in complex with the mature ligand^[68]^. This observation indicates that GDF8 can adopt both a latent state and an activated or ‘triggered’ state where the prodomain is still bound but GDF8 can nonetheless signal. Without activation, GDF8 retains a minor ability to stimulate signaling that is greatly enhanced following acid-activation. Structural analysis of the purified latent and acid-activated complexes using small angle X-ray scattering (SAXS) revealed that the acid-activated sample adopts a more open conformation than the latent prodomain-mature complex^[68]^. The idea of an open conformation was further supported by the crystal structure of the prodomain GDF8 complex^[69]^. Interestingly, point mutations have been identified in the prodomain that alleviate latency while the ligand is still bound to the prodomain^[68,70]^. These data support the concept that GDF8, and likely GDF11, exist in multiple molecular states including (i) a tightly inhibited latent state where the prodomain mimics a similar mechanism to TGF-ß latency; (ii) a triggered state where the prodomain is bound in a more “open” state and the ligand is active; (iii) a Tolloid processed state which is active but not defined molecularly; (iv) an apo-state where the ligand is active and free of binding partners; and (v) an antagonist bound state where the ligand is neutralized by extracellular antagonists [Figure 3]. While the total GDF8 or GDF11 does not appear to change with age, it will be important to understand if the populations of each ligand are altered with age. For example, an increase in the antagonist FSTL3 would have a direct impact on GDF8 and GDF11^[71]^.

### Tolloid proteases cleave the inhibitory prodomain of GDF11

Tolloid proteases (TLDs) are zinc-dependent metalloproteinases. The four mammalian TLDs include two alternative splice forms of the Bmp1 gene - bone morphogenetic protein 1 (Bmp1) and mammalian tolloid (mTLD), and two related proteins, tolloid-like 1 (TLL1) and TLL2^[72]^ [Figure 1 and 2]. TLDs have numerous proteolytic substrates that are essential for tissue patterning and extracellular matrix assembly^[73]^. TLDs also activate GDF8 and GDF11, both of which are secreted as latent precursors after cleavage by Furin which separates the large N-terminal prodomain from the C-terminal mature signaling domain^[74]^. Unlike other TGF-β superfamily proteins, the mature C-terminal domains of GDF8 and GDF11 remain tightly bound to their prodomains even after Furin cleavage and require TLD cleavage of the prodomain to convert from an inactive to a “triggered” state that is primed for subsequent activity of the mature, signaling-competent ligand^[75–77]^. As noted above, the proteolytic activation step appears to be essential for ligand function, as the introduction of TLD cleavage-resistant mutations into the GDF8 prodomain prevents ligand activation *in vitro*^[76]^ and *in vivo*^[78]^ and produces mice with significantly increased muscle mass, similar in phenotype to the phenotype elicited by genetic inactivation of GDF8 or administration of GDF8 inhibitors^[78]^, despite dramatic elevation of GDF8 levels in the sera of these animals. *In vivo* administration of exogenous TLD-resistant GDF8-unprocess-pre-procomplex likewise increases muscle mass; however, similar administration of the native GDF8-unprocess-pre-procomplex does not block GDF8 activity in this manner^[75]^. Thus, it appears that TLD-mediated proteolysis serves a key regulatory function for GDF8 and GDF11 protein activity, an observation that explains why measures of total GDF8 and GDF11 protein are not sufficient, alone, to assess the *in vivo*, functional activity of this signaling system.

### Recognition of human GDF11 genetic diseases

**Figure 4.**
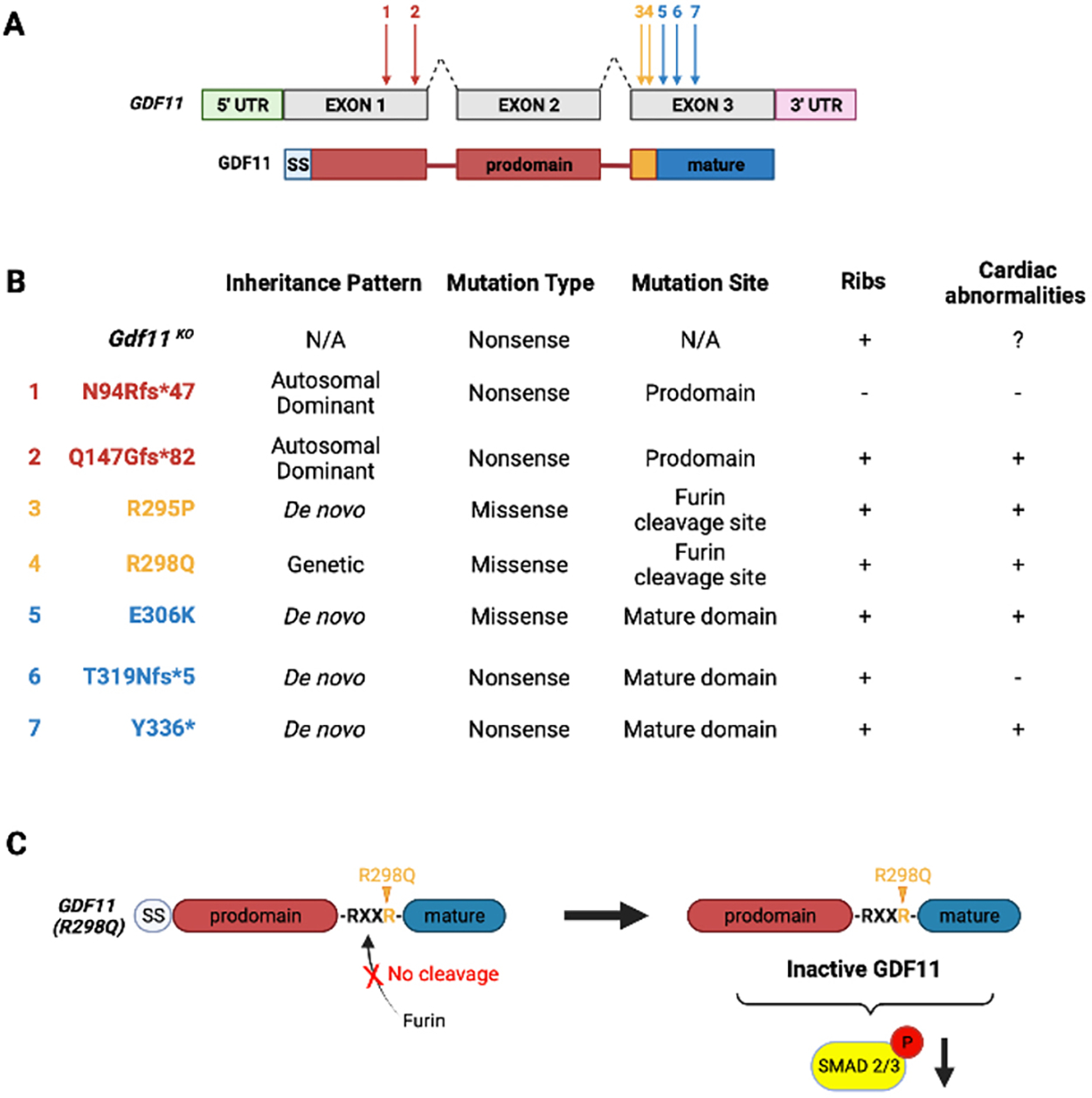
Human genetic diseases associated with GDF11 mutations. (A) 7 mutation sites have been identified in the GDF11 gene associated with several defects such as cleft palate and skeletal abnormalities. Mutations 1 and 2 are located in the prodomain, Mutations 3 and 4 in the Furin cleavage site, and Mutations 5–7 in the mature domain of GDF11. (B) Summary table of GDF11 mutations identified in humans. (+) indicates defects; (−) indicates no defect. + Ribs suggest skeleton defects. (C) Schematic depicting the functional impact of mutation 3, located in the Furin cleavage site, highlighting the importance of Furin cleavage for GDF11 activity.

An initial GDF11 mutation with a dominant inheritance pattern and variable penetrance has been reported to cause cleft lip/palate and rib/vertebral hypersegmentation as observed in Gdf11^−/−^ mice^[41]^. This mutation affects the RXXR motif that is essential for the cleavage by Furin and replaces the second Arginine with a Glutamine. The result of this mutation in humans is the absence of GDF11 cleavage and behaves as a dominant GDF11-loss-of-function variant^[79]^ [Figure 4]. The phenotype in this family was Cleft lip with or without cleft palate. Through the Undiagnosed Disease Network, multiple other loss-of-function heterozygous Gdf11 mutations were subsequently identified in patients with multi-system defects including neurological, cardiovascular, or ocular phenotypes^[6]^. These new data reveal the importance of GDF11 function in humans. Exploration of the Gnomad database suggests that mutations in GDF11 are infrequent (pLOF: pLI = 0.98 o/e = 0.06), but mutations in GDF11 are associated with cardiac diseases (HuGE score: 4.28).

## CONTROVERSIES

Prior work from our lab has identified GDF11 as a target of interest in aging-related dysfunction^[12,14,15,80,81]^. Some of our results were unexpected by the field, as they diverged from the previously established activities of GDF8^[82,83]^, and so our initial studies generated substantial discussion and controversy^[30,34,76,82,84]^. Here we describe some of the issues and outcomes surrounding the GDF11 debate (summarized in Table 1).

### Total circulating GDF11 does not decline with age

In 2013, we reported an important decline in systemic levels of GDF11 in aged compared to young mice^[15]^. This conclusion was based on results from a study using an aptamer-driven analysis of serum from young (2 months) *vs*. old (24 months) mice performed using Somalogics SomaMERs^[76]^, as well as Western blotting using a monoclonal antibody from Abcam, which, at the time, was reported to be specific for GDF11. Subsequent reports from the Glass laboratory at Novartis, and others including us^[30,85–87]^ revealed that the SomaMER and monoclonal antibody used in these initial studies cross-react with GDF8. Thus, while these studies were consistent with a reduction in the circulating pool of GDF11 + GDF8 in aged animals, our initial suggestion that systemic levels of GDF11 declined with aging was incorrect, as the GDF11 + GDF8 signal was dominated by GDF8, which has lower potency compared to GDF11 but circulates at substantially higher concentrations. A report by the Glass laboratory at Novartis argued that circulating levels of GDF11 might actually increase with age^[30]^; however, thus far, an increase in circulating levels of GDF11 has not been supported by mass spectrometry studies in mice or humans^[88]^. We are currently studying subforms of GDF11 and GDF8 in human blood, and it appears that specific subforms of GDF11 may change in abundance in an age-specific manner (unpublished observations).

### GDF11 and geronic effects

In prior publications^[12,14,15,80]^, we reported that supplementation of circulating GDF11 can reverse age-related deficits in multiple major organ systems, recapitulating many of the effects seen with heterochronic parabiosis. However, some subsequent publications have challenged the notion that GDF11 may be beneficial in aging (anti-geronic), suggesting that it also possesses potential “pro-geronic” actions. Such contradictory impacts on aging phenotypes are not dissimilar to those of other aging-relevant regulators, including IGF1^[89,90]^ metformin^[91]^, and rapamycin^[92]^, likely reflecting dose-dependent and context-specific functions. Still, a handful of studies^[30,32,84,93]^ have directly challenged our reports^[12,15]^ that supplementation of GDF11 can have beneficial effects on the heart and skeletal muscle. In this regard, as discussed above, it is important to note that subsequent work from our group demonstrated that at least some of the reported discrepancies relate to variability in the specific activity of commercially available recombinant GDF11 protein and to critical differences in experimental design^[76,80]^, and additional follow up studies from our labs^[80]^ as well as studies from independent research groups^[84,94–96]^, have confirmed the reproducibility of our published results. The most controversial aspects of GDF11’s potential geronic activities relate to its impact on skeletal muscle biology, which we address below.

### Effect of GDF11 supplementation on cardiac hypertrophy

Our studies published in 2013^[15]^ and 2015^[85]^ reported an anti-hypertrophic effect of rGDF11 administration by comparing heart weight-tibia length (HW/TL) ratio in treated and control aging mice, while a study by Smith *et al.* reported no effects on the heart^[32]^. As discussed in^[76]^, this disagreement appears to reflect dose-dependent effects of rGDF11 on cardiac mass, and an additional study revealed a positive influence of GDF11 on cardiac function and infarct size after cardiac injury in aged mice^[95]^. The potential benefits of GDF11 in cardiac hypertrophy^[97]^ have now been confirmed by multiple laboratories^[33,98–100]^.

### GDF11 and GDF8 - effects on skeletal muscle mass and regeneration

Initial studies from our labs indicated that rGDF11 supplementation reverses age-related muscle dysfunction and improves muscle strength, endurance, and regenerative potential in aged mice, with no discernable effects in young mice^[12]^. However, a subsequent paper from David Glass’s lab, then at Novartis, pursuing therapeutics that would antagonize GDF8 and GDF11 to treat age-related muscle dysfunction^[101–103]^, argued that supplementation with rGDF11 has no effect in aged mice and slows skeletal muscle repair in young mice^[104]^. As we discussed in a review published in 2016^[76]^, it is possible that these conflicting results arose from critical differences in experimental design, particularly in the use of different muscle injury models, and differences in the dosage and bioactivity of the particular rGDF11 molecule that was administered in each study. Specifically, while our studies used a cryoinjury model^[12]^, which causes limited damage to endogenous regenerative muscle stem cells, the Glass study^[104]^ used a more severe cardiotoxin (CTX) model, which ablates > 85% of satellite cells^[105,106]^. Given this severe depletion of muscle regenerative cells in the CTX model, it might have been predicted that rGDF11 would fail to enhance regeneration in CTX-injured aged mice. In contrast, we reported enhanced regeneration after single cryoinjury in rGDF11-supplemented aged animals, which retain a largely intact pool of muscle satellite cells. The Glass lab’s results in aged CTX-injured animals may reflect the severe depletion of regenerative stem cells in this model. This notion is further supported by data published by Sinha *et al.* demonstrating that when combined with satellite cell transplantation^[12]^, rGDF11 supplementation does indeed enhance regenerative myogenesis in aged, CTX-injured muscle. Importantly, as muscle injury in older humans more often resembles the focal damage induced by cryoinjury, as opposed to the full muscle necrosis caused by CTX, we believe additional experimentation will resolve the discrepancy in prior results and encourage continued investigation of GDF11 as a target for aging muscles.

### Exogenous GDF11 and toxicity

It is important to consider that the genetic loss of endogenous GDF11 and GDF8 function may not be the opposite of gain of function through exogenous mature ligands. There is extensive regulation of endogenous ligand activity through protease activation of the prodomains, for example. In addition, these ligands have several important endogenous inhibitors that can bind tightly and likely permanently inhibit signaling. Administration of mature GDF11 and GDF8 ligands bypasses the prodomain activation step and may lead to signaling before endogenous inhibitors can bind to the ligand. In the brain, endogenous GDF11 may have different functions depending on region, and exogenous GDF11 does not appear to appreciably penetrate the uninjured blood-brain barrier.

There also have been several reports suggesting that dosage of rGDF11 at very high levels in mice may drive muscle atrophy and fibrosis as well as death^[33,71]^. These data highlight the importance of understanding the biology of the GDF11 system, as articulated above. In particular, our studies clearly indicate a change in signaling when GDF11 is applied at very high levels, making it more “GDF8-like” and eliminating its normally pro-regenerative signaling activity. Thus, it is unsurprising that administration of rGDF11 at very high doses, which are neither physiologically nor therapeutically relevant, would produce Myostatin-like effects. Critically, such effects should not be taken as a true reflection of the normal biology of GDF11 *in vivo*, or its therapeutic potential. Indeed, data from our labs, and others, using much lower doses to achieve more modest increases in GDF11 levels have demonstrated a meaningful therapeutic window for GDF11 in numerous aging and disease models^[68,80]^.

## CONCLUSION AND PERSPECTIVES

As lifespan increases and the world’s population grows, the need to develop more effective approaches to treat heart disease and other age-associated dysfunctions is greater than ever. GDF11 biology may play roles in the progression of age-related diseases, but the simple concept that GDF11 levels decline with aging and can be replaced like thyroid hormone is incorrect. Despite conflicting reports over the potentially divergent functions of the two ligands and continuing controversy over GDF11 function and potential effects in age-associated organ dysfunction, GDF11 and GDF8 continue to be pursued as important disease targets for the development of possible therapeutics. The new human *GDF11* genetic diseases show that understanding GDF11 biology is important for humans, although the mutations may be rare. It is also unclear if GDF11 replacement could benefit patients with genetic loss of function mutations. For common diseases like coronary disease and other acquired diseases of aging, the role of GDF11 is unclear. The role of GDF11 measurements in human blood is incompletely defined, and a current conundrum is why mass spectrometry measurements of GDF11 in humans had not been revealing, while an aptamer measurement of GDF8/11 in humans was predictive of outcome in two moderate-size studies of coronary heart patients. More definitions of molecular mechanisms, including which target cells are activated, are needed to understand the effects of exogenous GDF11.

There are different major gaps in our understanding of how to leverage GDF11 as a potential therapeutic signaling molecule. First, the general coordination of cellular GDF11 signaling needs to be defined. There is a lack of spatial understanding regarding the series of processing, latency, and activation required for GDF11 signaling. Second, the best delivery mechanism for GDF11 therapy needs to be established. Current efforts have focused on injecting a bolus of mature GDF11. However, the half-life of GDF11 is ~12 h, and a significant amount of GDF11 needs to be injected, indicating a significant loss of the protein before it reaches its destination. Furthermore, injecting large amounts of GDF11 might deliver off-target effects, as was observed in mice with high doses of GDF11, which led to cachexia. Third, it is essential to understand how the recent discovery of GDF11 mutations impacts protein function and which methods of delivery (recombinant or viral expression) are capable of reintroducing GDF11 signaling in target cells.

## Figures and Tables

**Table 1.** Summary of the various controversies surrounding GDF11 - status and conclusions

Controversies	Status	Conclusion	References

Total circulating GDF11 during aging	Resolved	The total amount of circulating GDF8 (but not GDF11) declines with aging.	[31,16,86–89]
GDF11 and geronic effects	Partially resolved	Supplementation on rGDF11 can reverse age-related deficits in different organs (incompletely resolved in skeletal muscles)	[13,15,16,31,33,81, 85,97]
Cardiac hypertrophy	Resolved	Supplementation on rGDF11 reduces cardiac hypertrophy in aging	[16,33,34,87,103 106]
Exogenous GDF11 and toxicity	Resolved	Exogenous rGDF11 at high doses produces Myostatin-like effects	[34,72]

## Data Availability

No original data were generated for this review article.

## References

[1] López-Otín C, Blasco MA, Partridge L, Serrano M, Kroemer G. The hallmarks of aging. Cell 2013;153:1194–217. 23746838 10.1016/j.cell.2013.05.039 PMC3836174

[2] Ince S Nikolaos frangogiannis: preventing dangerous remodeling after myocardial infarction. Circ Res 2016;119:25–8. 27340269 10.1161/CIRCRESAHA.116.309141

[3] Dobaczewski M, Chen W, Frangogiannis NG. Transforming growth factor (TGF)-β signaling in cardiac remodeling. J Mol Cell Cardiol 2011;51:600–6. 21059352 10.1016/j.yjmcc.2010.10.033 PMC3072437

[4] Yue Y, Meng K, Pu Y, Zhang X. Transforming growth factor beta (TGF-β) mediates cardiac fibrosis and induces diabetic cardiomyopathy. Diabetes Res Clin Pract 2017;133:124–30. 28934669 10.1016/j.diabres.2017.08.018

[5] Batlle E, Massagué J. Transforming growth factor-β signaling in immunity and cancer. Immunity 2019;50:924–40. 30995507 10.1016/j.immuni.2019.03.024 PMC7507121

[6] Ravenscroft TA, Phillips JB, Fieg E, Heterozygous loss-of-function variants significantly expand the phenotypes associated with loss of GDF11. Genet Med 2021;23:1889–900. 34113007 10.1038/s41436-021-01216-8 PMC8487929

[7] Cui H, Kong Y, Zhang H. Oxidative stress, mitochondrial dysfunction, and aging. J Signal Transduct 2012;2012:1–13. 10.1155/2012/646354 PMC3184498 21977319

[8] Kikuchi K, Poss KD. Cardiac regenerative capacity and mechanisms. Annu Rev Cell Dev Biol 2012;28:719–41. 23057748 10.1146/annurev-cellbio-101011-155739 PMC3586268

[9] Vagnozzi RJ, Molkentin JD, Houser SR. New myocyte formation in the adult heart: endogenous sources and therapeutic implications. Circ Res 2018;123:159–76. 29976685 10.1161/CIRCRESAHA.118.311208 PMC6051429

[10] Conboy IM, Conboy MJ, Smythe GM, Rando TA. Notch-mediated restoration of regenerative potential to aged muscle. Science 2003;302:1575–7. 14645852 10.1126/science.1087573

[11] Carlson BM, Faulkner JA. Muscle transplantation between young and old rats: age of host determines recovery. Am J Physiol 1989;256:C1262–6. 2735398 10.1152/ajpcell.1989.256.6.C1262

[12] Sinha M, Jang YC, Oh J, Restoring systemic GDF11 levels reverses age-related dysfunction in mouse skeletal muscle. Science 2014;344:649–52. 24797481 10.1126/science.1251152 PMC4104429

[13] Sinha I, Sinha-hikim AP, Wagers AJ, Sinha-hikim I. Testosterone is essential for skeletal muscle growth in aged mice in a heterochronic parabiosis model. Cell Tissue Res 2014;357:815–21. 24859218 10.1007/s00441-014-1900-2 PMC4149819

[14] Katsimpardi L, Litterman NK, Schein PA, Vascular and neurogenic rejuvenation of the aging mouse brain by young systemic factors. Science 2014;344:630–4. 24797482 10.1126/science.1251141 PMC4123747

[15] Loffredo FS, Steinhauser ML, Jay SM, Growth differentiation factor 11 is a circulating factor that reverses age-related cardiac hypertrophy. Cell 2013;153:828–39. 23663781 10.1016/j.cell.2013.04.015 PMC3677132

[16] Ruckh JM, Zhao JW, Shadrach JL, Rejuvenation of regeneration in the aging central nervous system. Cell Stem Cell 2012;10:96–103. 22226359 10.1016/j.stem.2011.11.019 PMC3714794

[17] Conboy IM, Conboy MJ, Wagers AJ, Girma ER, Weissman IL, Rando TA. Rejuvenation of aged progenitor cells by exposure to a young systemic environment. Nature 2005;433:760–4. 15716955 10.1038/nature03260

[18] Miron VE, Boyd A, Zhao JW, M2 microglia and macrophages drive oligodendrocyte differentiation during CNS remyelination. Nat Neurosci 2013;16:1211–8. 23872599 10.1038/nn.3469 PMC3977045

[19] Brack AS, Conboy MJ, Roy S, Increased Wnt signaling during aging alters muscle stem cell fate and increases fibrosis. Science 2007;317:807–10. 17690295 10.1126/science.1144090

[20] Villeda SA, Plambeck KE, Middeldorp J, Young blood reverses age-related impairments in cognitive function and synaptic plasticity in mice. Nat Med 2014;20:659–63. 24793238 10.1038/nm.3569 PMC4224436

[21] Salpeter SJ, Khalaileh A, Weinberg-Corem N, Ziv O, Glaser B, Dor Y. Systemic regulation of the age-related decline of pancreatic β-cell replication. Diabetes 2013;62:2843–8. 23630298 10.2337/db13-0160 PMC3717843

[22] Baht GS, Silkstone D, Vi L, Exposure to a youthful circulaton rejuvenates bone repair through modulation of β-catenin. Nat Commun 2015;6:7131. 25988592 10.1038/ncomms8131 PMC4479006

[23] Elabd C, Cousin W, Upadhyayula P, Oxytocin is an age-specific circulating hormone that is necessary for muscle maintenance and regeneration. Nat Commun 2014;5:4082. 24915299 10.1038/ncomms5082 PMC4512838

[24] Smith LK, He Y, Park JS, β2-microglobulin is a systemic pro-aging factor that impairs cognitive function and neurogenesis. Nat Med 2015;21:932–7. 26147761 10.1038/nm.3898 PMC4529371

[25] Brack AS, Conboy MJ, Roy S, Increased wnt signaling during aging alters muscle stem cell fate and increases fibrosis. Science 2007;317:807–10. 17690295 10.1126/science.1144090

[26] Bieri G, Schroer AB, Villeda SA. Blood-to-brain communication in aging and rejuvenation. Nat Neurosci 2023;26:379–93. 36646876 10.1038/s41593-022-01238-8

[27] Hsiao YT, Shimizu I, Yoshida Y, Minamino T. Role of circulating molecules in age-related cardiovascular and metabolic disorders. Inflamm Regen 2022;42:2. 35012677 10.1186/s41232-021-00187-2 PMC8744343

[28] Pluvinage JV, Wyss-Coray T. Systemic factors as mediators of brain homeostasis, ageing and neurodegeneration. Nat Rev Neurosci 2020;21:93–102. 31913356 10.1038/s41583-019-0255-9

[29] Rando TA, Jones DL. Regeneration, rejuvenation, and replacement: turning back the clock on tissue aging. Cold Spring Harb Perspect Biol 2021;13:a040907. 34187808 10.1101/cshperspect.a040907 PMC8411956

[30] Egerman MA, Cadena SM, Gilbert JA, GDF11 increases with age and inhibits skeletal muscle regeneration. Cell Metab 2015;22:164–74. 26001423 10.1016/j.cmet.2015.05.010 PMC4497834

[31] Peng L, Gagliano-Jucá T, Pencina KM, Age trends in growth and differentiation factor-11 and myostatin levels in healthy men, and differential response to testosterone, measured using liquid chromatography-tandem mass spectrometry. J Gerontol A Biol Sci Med Sci 2022;77:763–9. 34037752 10.1093/gerona/glab146 PMC8974345

[32] Smith SC, Zhang X, Zhang X, GDF11 does not rescue aging-related pathological hypertrophy. Circ Res 2015;117:926–32. 26383970 10.1161/CIRCRESAHA.115.307527 PMC4636963

[33] Harper SC, Johnson J, Borghetti G, GDF11 decreases pressure overload-induced hypertrophy, but can cause severe cachexia and premature death. Circ Res 2018;123:1220–31. 30571461 10.1161/CIRCRESAHA.118.312955 PMC6309347

[34] Hammers DW, Merscham-Banda M, Hsiao JY, Engst S, Hartman JJ, Sweeney HL. Supraphysiological levels of GDF11 induce striated muscle atrophy. EMBO Mol Med 2017;9:531–44. 28270449 10.15252/emmm.201607231 PMC5376753

[35] Jones JE, Cadena SM, Gong C, Supraphysiologic administration of GDF11 induces cachexia in part by upregulating GDF15. Cell Rep 2018;22:1522–30. 29425507 10.1016/j.celrep.2018.01.044

[36] McPherron AC, Lawler AM, Lee SJ. Regulation of skeletal muscle mass in mice by a new TGF-beta superfamily member. Nature 1997;387:83–90. 9139826 10.1038/387083a0

[37] McPherron AC, Lee SJ. Double muscling in cattle due to mutations in the myostatin gene. Proc Natl Acad Sci USA 1997;94:12457–61. 9356471 10.1073/pnas.94.23.12457 PMC24998

[38] Mosher DS, Quignon P, Bustamante CD, A mutation in the myostatin gene increases muscle mass and enhances racing performance in heterozygote dogs. PLoS Genet 2007;3:e79. 17530926 10.1371/journal.pgen.0030079 PMC1877876

[39] Medeiros EF, Phelps MP, Fuentes FD, Bradley TM. Overexpression of follistatin in trout stimulates increased muscling. Am J Physiol Regul Integr Comp Physiol 2009;297:R235–42. 19474387 10.1152/ajpregu.91020.2008

[40] McPherron AC, Lawler AM, Lee SJ. Regulation of anterior/posterior patterning of the axial skeleton by growth/differentiation factor 11. Nat Genet 1999;22:260–4. 10391213 10.1038/10320

[41] McPherron AC, Huynh TV, Lee SJ. Redundancy of myostatin and growth/differentiation factor 11 function. BMC Dev Biol 2009;9:24. 19298661 10.1186/1471-213X-9-24 PMC2666675

[42] Harmon EB, Apelqvist AA, Smart NG, Gu X, Osborne DH, Kim SK. GDF11 modulates NGN3^+^ islet progenitor cell number and promotes beta-cell differentiation in pancreas development. Development 2004;131:6163–74. 15548585 10.1242/dev.01535

[43] JP L The function of growth/differentiation factor 11 (Gdf11) in rostrocaudal patterning of the developing spinal cord. Development 2006;133:2865074. 10.1242/dev.02478 16790475

[44] Wu HH, Ivkovic S, Murray RC, Autoregulation of neurogenesis by GDF11. Neuron 2003;37:197–207. 12546816 10.1016/s0896-6273(02)01172-8

[45] Kim J, Wu HH, Lander AD, Lyons KM, Matzuk MM, Calof AL. GDF11 controls the timing of progenitor cell competence in developing retina. Science 2005;308:1927–30. 15976303 10.1126/science.1110175

[46] Li Y, Choi WJ, Wei W, Aging-associated changes in cerebral vasculature and blood flow as determined by quantitative optical coherence tomography angiography. Neurobiol Aging 2018;70:148–59. 30007164 10.1016/j.neurobiolaging.2018.06.017 PMC6119107

[47] Bullitt E, Zeng D, Mortamet B, The effects of healthy aging on intracerebral blood vessels visualized by magnetic resonance angiography. Neurobiol Aging 2010;31:290–300. 18471935 10.1016/j.neurobiolaging.2008.03.022 PMC2806428

[48] Graff BJ, Payne SJ, El-Bouri WK. The ageing brain: investigating the role of age in changes to the human cerebral microvasculature with an in silico model. Front Aging Neurosci 2021;13:632521. 34421568 10.3389/fnagi.2021.632521 PMC8374868

[49] Knox EG, Aburto MR, Clarke G, Cryan JF, O’Driscoll CM. The blood-brain barrier in aging and neurodegeneration. Mol Psychiatry 2022;27:2659–73. 35361905 10.1038/s41380-022-01511-z PMC9156404

[50] Chen MB, Yang AC, Yousef H, Brain endothelial cells are exquisite sensors of age-related circulatory cues. Cell Rep 2020;30:4418–32.e4. 32234477 10.1016/j.celrep.2020.03.012 PMC7292569

[51] Ximerakis M, Lipnick SL, Innes BT, Single-cell transcriptomic profiling of the aging mouse brain. Nat Neurosci 2019;22:1696–708. 31551601 10.1038/s41593-019-0491-3

[52] Middeldorp J, Lehallier B, Villeda SA, Preclinical assessment of young blood plasma for alzheimer disease. JAMA Neurol 2016;73:1325–33. 27598869 10.1001/jamaneurol.2016.3185 PMC5172595

[53] Villeda SA, Luo J, Mosher KI, The ageing systemic milieu negatively regulates neurogenesis and cognitive function. Nature 2011;477:90–4. 21886162 10.1038/nature10357 PMC3170097

[54] Ximerakis M, Holton KM, Giadone RM, Heterochronic parabiosis reprograms the mouse brain transcriptome by shifting aging signatures in multiple cell types. Nat Aging 2023;3:327–45. 37118429 10.1038/s43587-023-00373-6 PMC10154248

[55] Katsimpardi L, Litterman NK, Schein PA, Vascular and neurogenic rejuvenation of the aging mouse brain by young systemic factors. Science 2014;344:630–4. 24797482 10.1126/science.1251141 PMC4123747

[56] He Y, Zhang H, Yung A, ALK5-dependent TGF-β signaling is a major determinant of late-stage adult neurogenesis. Nat Neurosci 2014;17:943–52. 24859199 10.1038/nn.3732 PMC4096284

[57] Shen Q, Goderie SK, Jin L, Endothelial cells stimulate self-renewal and expand neurogenesis of neural stem cells. Science 2004;304:1338–40. 15060285 10.1126/science.1095505

[58] Kokovay E, Goderie S, Wang Y, Adult SVZ lineage cells home to and leave the vascular niche via differential responses to SDF1/CXCR4 signaling. Cell Stem Cell 2010;7:163–73. 20682445 10.1016/j.stem.2010.05.019 PMC2916873

[59] Kawauchi S, Kim J, Santos R, Wu HH, Lander AD, Calof AL. Foxg1 promotes olfactory neurogenesis by antagonizing Gdf11. Development 2009;136:1453–64. 19297409 10.1242/dev.034967 PMC2674256

[60] Mayweather BA, Buchanan SM, Rubin LL. GDF11 expressed in the adult brain negatively regulates hippocampal neurogenesis. Mol Brain 2021;14:134. 34488822 10.1186/s13041-021-00845-z PMC8422669

[61] Zhang W, Guo Y, Li B, GDF11 rejuvenates cerebrovascular structure and function in an animal model of alzheimer’s disease. J Alzheimers Dis 2018;62:807–19. 29480172 10.3233/JAD-170474

[62] Ma J, Zhang L, Niu T, Growth differentiation factor 11 improves neurobehavioral recovery and stimulates angiogenesis in rats subjected to cerebral ischemia/reperfusion. Brain Res Bull 2018;139:38–47. 29432795 10.1016/j.brainresbull.2018.02.011

[63] Hudobenko J, Ganesh BP, Jiang J, Growth differentiation factor-11 supplementation improves survival and promotes recovery after ischemic stroke in aged mice. Aging 2020;12:8049–66. 32365331 10.18632/aging.103122 PMC7244081

[64] Walker RG, Czepnik M, Goebel EJ, Structural basis for potency differences between GDF8 and GDF11. BMC Biol 2017;15:19. 28257634 10.1186/s12915-017-0350-1 PMC5336696

[65] Lian J, Walker RG, D’Amico A, Functional substitutions of amino acids that differ between GDF11 and GDF8 impact skeletal development and skeletal muscle. Life Sci Alliance 2023;6:e202201662. 36631218 10.26508/lsa.202201662 PMC9834663

[66] Lee SJ, Lehar A, Rydzik R, Functional replacement of myostatin with GDF-11 in the germline of mice. Skelet Muscle 2022;12:7. 35287700 10.1186/s13395-022-00290-z PMC8922734

[67] Rebbapragada A, Benchabane H, Wrana JL, Celeste AJ, Attisano L. Myostatin signals through a transforming growth factor β-like signaling pathway to block adipogenesis. Mol Cell Biol 2003;23:7230–42. 14517293 10.1128/MCB.23.20.7230-7242.2003 PMC230332

[68] Walker RG, McCoy JC, Czepnik M, Molecular characterization of latent GDF8 reveals mechanisms of activation. Proc Natl Acad Sci USA 2018;115:E866–75. 29348202 10.1073/pnas.1714622115 PMC5798348

[69] Le VQ, Iacob RE, Tian Y, Tolloid cleavage activates latent GDF8 by priming the pro-complex for dissociation. EMBO J 2018;37:384–97. 29343545 10.15252/embj.201797931 PMC5793799

[70] McCoy JC, Goebel EJ, Thompson TB. Characterization of tolloid-mediated cleavage of the GDF8 procomplex. Biochem J 2021;478:1733–47. 33876824 10.1042/BCJ20210054 PMC8670536

[71] Roh JD, Hobson R, Chaudhari V, Activin type II receptor signaling in cardiac aging and heart failure. Sci Transl Med 2019:11. 10.1126/scitranslmed.aau8680 PMC7124007 30842316

[72] Goff S, Hulmes DJS, Moali C, BMP-1/tolloid-like proteinases synchronize matrix assembly with growth factor activation to promote morphogenesis and tissue remodeling. Matrix Biol 2015;44–46:14–23. 10.1016/j.matbio.2015.02.006 25701650

[73] Troilo H, Bayley CP, Barrett AL, Lockhart-Cairns MP, Jowitt TA, Baldock C. Mammalian tolloid proteinases: role in growth factor signalling. FEBS Lett 2016;590:2398–407. 27391803 10.1002/1873-3468.12287 PMC4988381

[74] Lee SJ, McPherron AC. Regulation of myostatin activity and muscle growth. Proc Natl Acad Sci USA 2001;98:9306–11. 11459935 10.1073/pnas.151270098 PMC55416

[75] Wolfman NM, McPherron AC, Pappano WN, Activation of latent myostatin by the BMP-1/tolloid family of metalloproteinases. Proc Natl Acad Sci USA 2003;100:15842–6. 14671324 10.1073/pnas.2534946100 PMC307655

[76] Walker RG, Poggioli T, Katsimpardi L, Biochemistry and biology of GDF11 and myostatin: similarities, differences, and questions for future investigation. Circ Res 2016;118:1125–41;discussion 1142. 27034275 10.1161/CIRCRESAHA.116.308391 PMC4818972

[77] Ge G, Hopkins DR, Ho WB, Greenspan DS. GDF11 forms a bone morphogenetic protein 1-activated latent complex that can modulate nerve growth factor-induced differentiation of PC12 cells. Mol Cell Biol 2005;25:5846–58. 15988002 10.1128/MCB.25.14.5846-5858.2005 PMC1168807

[78] Lee SJ. Genetic analysis of the role of proteolysis in the activation of latent myostatin. PLoS One 2008;3:e1628. 18286185 10.1371/journal.pone.0001628 PMC2237902

[79] Cox TC, Lidral AC, McCoy JC, Mutations in GDF11 and the extracellular antagonist, Follistatin, as a likely cause of Mendelian forms of orofacial clefting in humans. Hum Mutat 2019;40:1813–25. 31215115 10.1002/humu.23793 PMC6764866

[80] Poggioli T, Vujic A, Yang P, Circulating growth differentiation factor 11/8 levels decline with age. Circ Res 2016;118:29–37. 26489925 10.1161/CIRCRESAHA.115.307521 PMC4748736

[81] Ozek C, Krolewski RC, Buchanan SM, Rubin LL. Growth differentiation factor 11 treatment leads to neuronal and vascular improvements in the hippocampus of aged mice. Sci Rep 2018;8:17293. 30470794 10.1038/s41598-018-35716-6 PMC6251885

[82] Glass DJ. Elevated GDF11 is a risk factor for age-related frailty and disease in humans. Cell Metab 2016;24:7–8. 27411004 10.1016/j.cmet.2016.06.017

[83] Rodgers BD. The immateriality of circulating GDF11. Circ Res 2016;118:1472–4. 27174947 10.1161/CIRCRESAHA.116.308478

[84] Zhou Y, Sharma N, Dukes D, GDF11 treatment attenuates the recovery of skeletal muscle function after injury in older rats. AAPS J 2017;19:431–7. 27924614 10.1208/s12248-016-0024-x

[85] Poggioli T, Vujic A, Yang P, Circulating growth differentiation factor 11/8 levels decline with age. Circ Res 2016;118:29–37. 26489925 10.1161/CIRCRESAHA.115.307521 PMC4748736

[86] Olson KA, Beatty AL, Heidecker B, Association of growth differentiation factor 11/8, putative anti-ageing factor, with cardiovascular outcomes and overall mortality in humans: analysis of the heart and soul and HUNT3 cohorts. Eur Heart J 2015;36:3426–34. 26294790 10.1093/eurheartj/ehv385 PMC4685178

[87] Hathout Y, Brody E, Clemens PR, Large-scale serum protein biomarker discovery in Duchenne muscular dystrophy. Proc Natl Acad Sci USA 2015;112:7153–8. 26039989 10.1073/pnas.1507719112 PMC4466703

[88] Schafer MJ, Atkinson EJ, Vanderboom PM, Quantification of GDF11 and myostatin in human aging and cardiovascular disease. Cell Metab 2016;23:1207–15. 27304512 10.1016/j.cmet.2016.05.023 PMC4913514

[89] Xu J, Gontier G, Chaker Z, Lacube P, Dupont J, Holzenberger M. Longevity effect of IGF-1R^+/−^ mutation depends on genetic background-specific receptor activation. Aging Cell 2014;13:19–28. 23898955 10.1111/acel.12145 PMC4326867

[90] Conover CA. PAPP-A: a new anti-aging target? Aging Cell 2010;9:942–6. 20854420 10.1111/j.1474-9726.2010.00630.x PMC3047409

[91] Mohammed I, Hollenberg MD, Ding H, Triggle CR. A critical review of the evidence that metformin is a putative anti-aging drug that enhances healthspan and extends lifespan. Front Endocrinol 2021;12:718942. 10.3389/fendo.2021.718942 PMC8374068 34421827

[92] Blagosklonny MV. Cell senescence, rapamycin and hyperfunction theory of aging. Cell Cycle 2022;21:1456–67. 35358003 10.1080/15384101.2022.2054636 PMC9278457

[93] Rodgers BD, Eldridge JA. Reduced circulating GDF11 is unlikely responsible for age-dependent changes in mouse heart, muscle, and brain. Endocrinology 2015;156:3885–8. 26372181 10.1210/en.2015-1628

[94] Demontis F, Patel VK, Swindell WR, Perrimon N. Intertissue control of the nucleolus via a myokine-dependent longevity pathway. Cell Rep 2014;7:1481–94. 24882005 10.1016/j.celrep.2014.05.001 PMC4125979

[95] Du GQ, Shao ZB, Wu J, Targeted myocardial delivery of GDF11 gene rejuvenates the aged mouse heart and enhances myocardial regeneration after ischemia-reperfusion injury. Basic Res Cardiol 2017;112:7. 28004242 10.1007/s00395-016-0593-y

[96] Ma S, Upneja A, Galecki A, Cell culture-based profiling across mammals reveals DNA repair and metabolism as determinants of species longevity. eLife 2016;5:e19130. 27874830 10.7554/eLife.19130 PMC5148604

[97] Duran J, Troncoso MF, Lagos D, Ramos S, Marin G, Estrada M. GDF11 modulates Ca^2+^-dependent Smad2/3 signaling to prevent cardiomyocyte hypertrophy. Int J Mol Sci 2018;19:1508. 29783655 10.3390/ijms19051508 PMC5983757

[98] Zhu HZ, Zhang LY, Zhai ME, GDF11 Alleviates pathological myocardial remodeling in diabetic cardiomyopathy through SIRT1-dependent regulation of oxidative stress and apoptosis. Front Cell Dev Biol 2021;9:686848. 34262905 10.3389/fcell.2021.686848 PMC8273395

[99] Zhang C, Wang Y, Ge Z, GDF11 attenuated ANG II-induced hypertrophic cardiomyopathy and expression of ANP, BNP and Beta-MHC through down- regulating CCL11 in mice. Curr Mol Med 2018;18:661–71. 30714521 10.2174/1566524019666190204112753

[100] Garrido-Moreno V, Díaz-Vegas A, López-Crisosto C, GDF-11 prevents cardiomyocyte hypertrophy by maintaining the sarcoplasmic reticulum-mitochondria communication. Pharmacol Res 2019;146:104273. 31096010 10.1016/j.phrs.2019.104273

[101] Amato AA, Sivakumar K, Goyal N, Treatment of sporadic inclusion body myositis with bimagrumab. Neurology 2014;83:2239–46. 25381300 10.1212/WNL.0000000000001070 PMC4277670

[102] Lach-Trifilieff E, Minetti GC, Sheppard K, An antibody blocking activin type II receptors induces strong skeletal muscle hypertrophy and protects from atrophy. Mol Cell Biol 2014;34:606–18. 24298022 10.1128/MCB.01307-13 PMC3911487

[103] Morvan F, Rondeau JM, Zou C, Blockade of activin type II receptors with a dual anti-ActRIIA/IIB antibody is critical to promote maximal skeletal muscle hypertrophy. Proc Natl Acad Sci USA 2017;114:12448–53. 29109273 10.1073/pnas.1707925114 PMC5703284

[104] Egerman MA, Cadena SM, Gilbert JA, GDF11 increases with age and inhibits skeletal muscle regeneration. Cell Metab 2015;22:164–74. 26001423 10.1016/j.cmet.2015.05.010 PMC4497834

[105] Polesskaya A, Seale P, Rudnicki MA. Wnt signaling induces the myogenic specification of resident CD45^+^ adult stem cells during muscle regeneration. Cell 2003;113:841–52. 12837243 10.1016/s0092-8674(03)00437-9

[106] Gayraud-Morel B, Chrétien F, Tajbakhsh S. Skeletal muscle as a paradigm for regenerative biology and medicine. Regen Med 2009;4:293–319. 19317647 10.2217/17460751.4.2.293

